# Nanometric Surficial Reflectors: Achieving High‐Performance and High‐Throughput Structural Coloration Through Modulation‐Assisted Machining

**DOI:** 10.1002/advs.202506162

**Published:** 2025-11-05

**Authors:** Yaoke Wang, Malachi Landis, Ping Guo

**Affiliations:** ^1^ Northwestern University L286, 2145 Sheridan Road Evanston, IL 60208 USA

**Keywords:** diffraction, modulation‐assisted machining, structural coloration, wavelength reflector

## Abstract

Structural coloration, which generates color through physical structures, has broad applications but often faces challenges in achieving high color performance while being scalable for mass production. In this study, a novel single‐layer wavelength‐selective reflector capable of producing a broad color gamut with local angle independence is introduced, fabricated using a modulation‐assisted ultra‐precision machining process. The structure features multi‐level submicron steps that sequentially filter light of different wavelengths, overcoming the limitations of conventional periodic structures that suffer from narrow color gamut and angle‐dependent color shifts. This approach integrates a purely mechanical machining process, shaping reflectors in a single step using a precisely controlled modulation trajectory. This enables efficient, scalable fabrication while preserving the desired structural characteristics at the nanoscale. Color performance comparable to the sRGB space is demonstrated and full‐color images are successfully rendered on metallic surfaces. The proposed reflector design provides a new pathway to scalable production of vibrant, angle‐independent structural colors with potential applications in anti‐counterfeiting, data storage, and high‐quality coloration.

## Graphical abstract

A novel approach is proposed to structural coloration overcomes the challenge of achieving high performance, color consistency, and scalability. This work introduces a single‐layer, wavelength‐selective reflector fabricated by a scalable, modulation‐assisted machining process. The method generates a broad, angle‐independent color gamut, enabling the rendering of full‐color, high‐quality images on metallic surfaces.

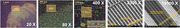

## 1 Introduction

**Figure 1:**
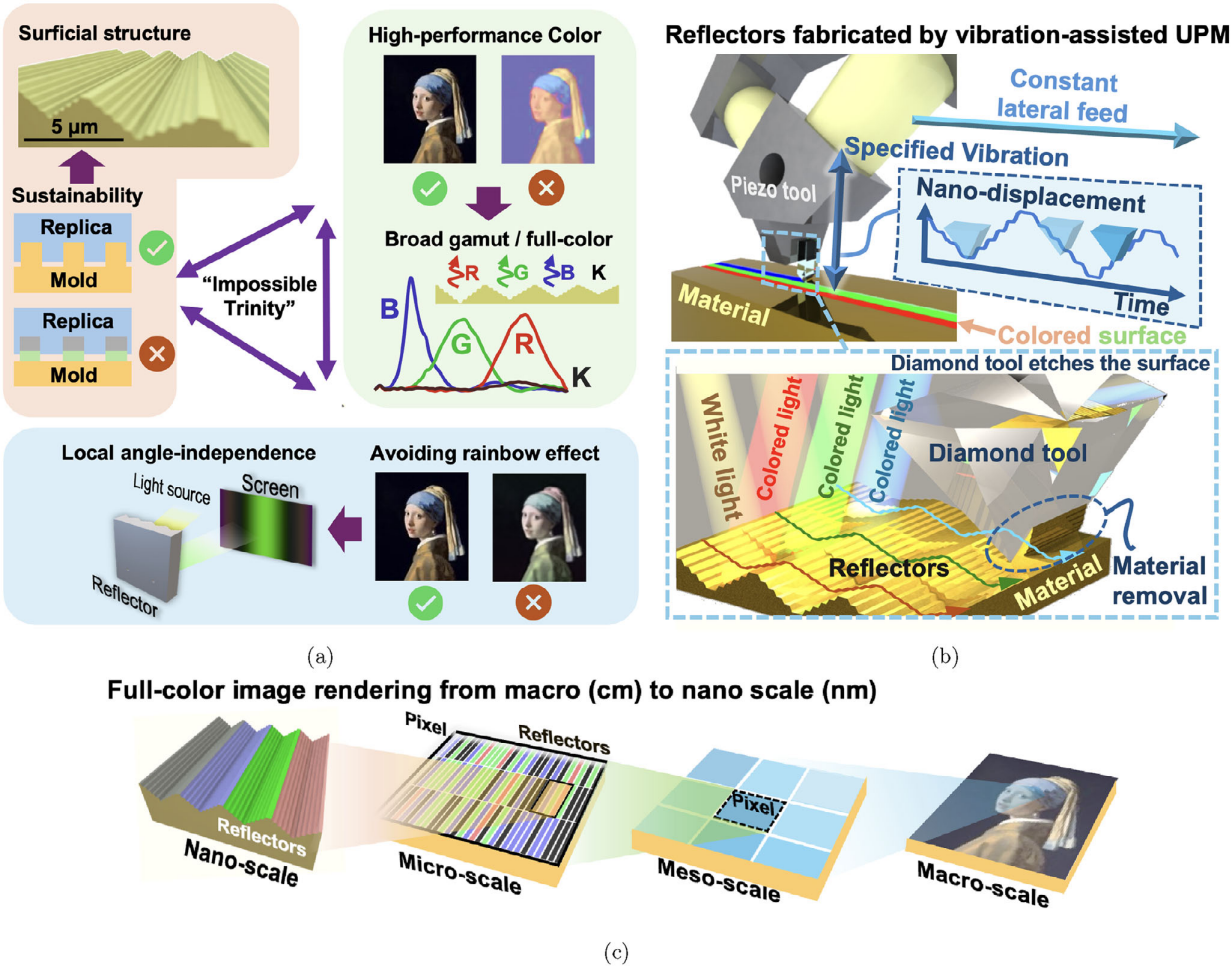
a) “Impossible trinity” of structural coloration, illustrating the trade‐offs between high‐performance color presentation, consistent color output, and scalability for low‐cost mass production; b) proposed solution of wavelength‐selective reflectors fabricated by modulation‐assisted machining; c) full‐color image rendering using the wavelength‐selective reflectors.

Structural coloration occurs when color is produced through wavelength‐selective lightwave interference or light‐matter interaction, created by specific physical structures.^[^ ^1^ ^]^ Artificial structural coloration techniques have been extensively developed over the past decades, leveraging subwavelength‐scale nanostructures to manipulate light and offering a promising alternative to traditional pigment‐based coloration.^[^ ^2^ ^]^ These methods are now applied across a variety of fields, including information storage,^[^ ^3^, ^4^ ^]^ anti‐counterfeiting,^[^ ^5^, ^6^ ^]^ and sensing technologies.^[^ ^7^, ^8^, ^9^, ^10^ ^]^ However, these applications demand a combination of high‐performance color presentation, consistent color output, and scalability for low‐cost mass production. Existing techniques typically optimize only one or two of the three requirements. Achieving all three simultaneously, including high performance, color consistency, and scalability, remains an “impossible trinity” (**Figure** **1**).

In this study, we address this challenge by introducing a surficial wavelength‐selective reflector, capable of producing high‐performance, angle‐independent color through an efficient and scalable modulation‐assisted machining process. Our approach not only challenges the limitations of traditional structural coloration techniques but also introduces a pathway to industrial‐scale production of full‐spectrum, vibrant colors on metallic surfaces.

Various techniques have been developed to achieve high‐performance structural coloration, but each faces limitations in scalability and consistency. Desired optical resonances, including thin‐film resonance,^[^ ^11^, ^12^ ^]^ guided‐mode resonance,^[^ ^13^ ^]^ and surface plasmon resonance,^[^ ^14^, ^15^ ^]^ can be excited by strategically manipulating the dielectric properties and structural layouts. These approaches to achieve optical resonances produce a controlled wavelength‐selective response.^[^ ^16^ ^]^ The all‐dielectric metasurfaces, for example, enable an extraordinarily wide color gamut.^[^ ^4^, ^17^ ^]^ Besides, by constructing laser‐induced,^[^ ^18^ ^]^ multi‐level,^[^ ^19^ ^]^ or particle‐based structures,^[^ ^20^, ^21^, ^22^ ^]^ the global angle‐independence can be achieved, ensuring color consistency across varying perspectives. Yet, these methods remain constrained by their reliance on multi‐layer or cavity‐based designs, limiting scalability for mass production. Scalability for high‐volume and large‐area production of structural colors remains challenging, leading to sustainability issues.^[^ ^23^ ^]^

As a result, single‐layer surficial structures have been explored, offering greater compatibility with large‐scale manufacturing processes such as roll‐to‐roll production and hot embossing.^[^ ^24^ ^]^

The predominant approaches for achieving single‐layer surficial structural coloration rely on periodic structures, which are fabricated through energy‐based or mechanical‐machining processes.^[^ ^25^, ^26^, ^27^, ^28^, ^29^, ^30^, ^31^, ^32^ ^]^ However, the dependence on periodic structures means that color is produced via spatial modulation rather than self‐resonance.^[^ ^33^ ^]^ This results in angle‐dependent color shifts and aliasing effects from different viewing angles, typically leading to a narrow color gamut and a noticeable rainbow effect. Moreover, the dependence on periodic structures limits the presented color to showing variation in hue only, falling short of rendering full‐spectrum colors. To completely eliminate the rainbow effect from a single perspective, a “local” angle‐independent performance is required, ensuring consistent color within a limited angular range. Existing solutions that aim to address either full‐color rendering^[^ ^34^ ^]^ or angle independence^[^ ^24^ ^]^ fall short of simultaneously achieving both.

Here, we propose a wavelength‐selective reflector for local angle‐independent full‐color presentation. The major contribution lies in the combination of a single‐layer structure generated through optical optimization and a purely mechanical fabrication approach, enabling both design flexibility and nanometer‐level accuracy.

On the optical design side, a multi‐level surface profile is optimized using a genetic algorithm to achieve efficient color filtering through 0th‐order diffraction. Unlike previous works that focused on theoretical profiles with extreme complexity for specific colors,^[^ ^35^ ^]^ our design resolves the “impossible trinity” by achieving high color performance, local angle independence, and compatibility with scalable manufacturing processes. At the same time, the resulting structure functions as a “surficial Bragg reflector,” where the reflected wavelength is primarily determined by the step height of the structure, making depth precision essential.

To fabricate the reflector with extreme depth accuracy, we adopt modulation‐assisted ultra‐precision machining (UPM), as illustrated in Figure 1, to directly realize the nanometric surface profiles. In this method, the cutting tool follows a precisely controlled vibration trajectory generated by a piezoelectric actuator, much like vinyl record cutting, allowing submicron features to be machined with nanometer‐level accuracy. While lithography‐based techniques,^[^ ^36^, ^37^ ^]^ such as multistep,^[^ ^38^ ^]^ or grayscale exposure^[^ ^39^, ^40^, ^41^ ^]^ offer alternative fabrication routes, they are limited by nonlinear exposure–depth relationships and photoresist variability. In contrast, precision machining avoids these issues and enables the direct production of metallic molds,^[^ ^42^, ^43^ ^]^ which can be readily integrated into mass replication techniques such as hot embossing^[^ ^44^ ^]^ and injection molding.^[^ ^45^ ^]^ Our approach thus provides a practical and scalable solution for fabricating structurally colored surfaces with complex profiles and nanometer‐level accuracy.

Our fabricated reflector structures demonstrate a gamut that covers most of the sRGB color space, with a local angle‐independent range exceeding 5° when subjected to varying incident angles and perspectives. We demonstrate full‐color image rendering in the RGB color space on metallic surfaces, exhibiting a consistent color tone without the unwanted rainbow effect. These rendered images consist of hierarchical structures at different scales, offering additional coding capabilities for applications such as anti‐counterfeiting and data storage. Our proposed approach, surficial wavelength‐selective reflectors combined with modulation‐assisted ultra‐precision machining, offers a promising route toward scalable, high‐performance structural coloration, bridging the gap between lab‐scale precision and industrial‐level mass production, thus paving the way for numerous practical applications for structural coloration.

## 2 Results and Discussion

### 2.1 Manufacturable Design for Surficial Wavelength‐Selective Reflectors

**Figure 2:**
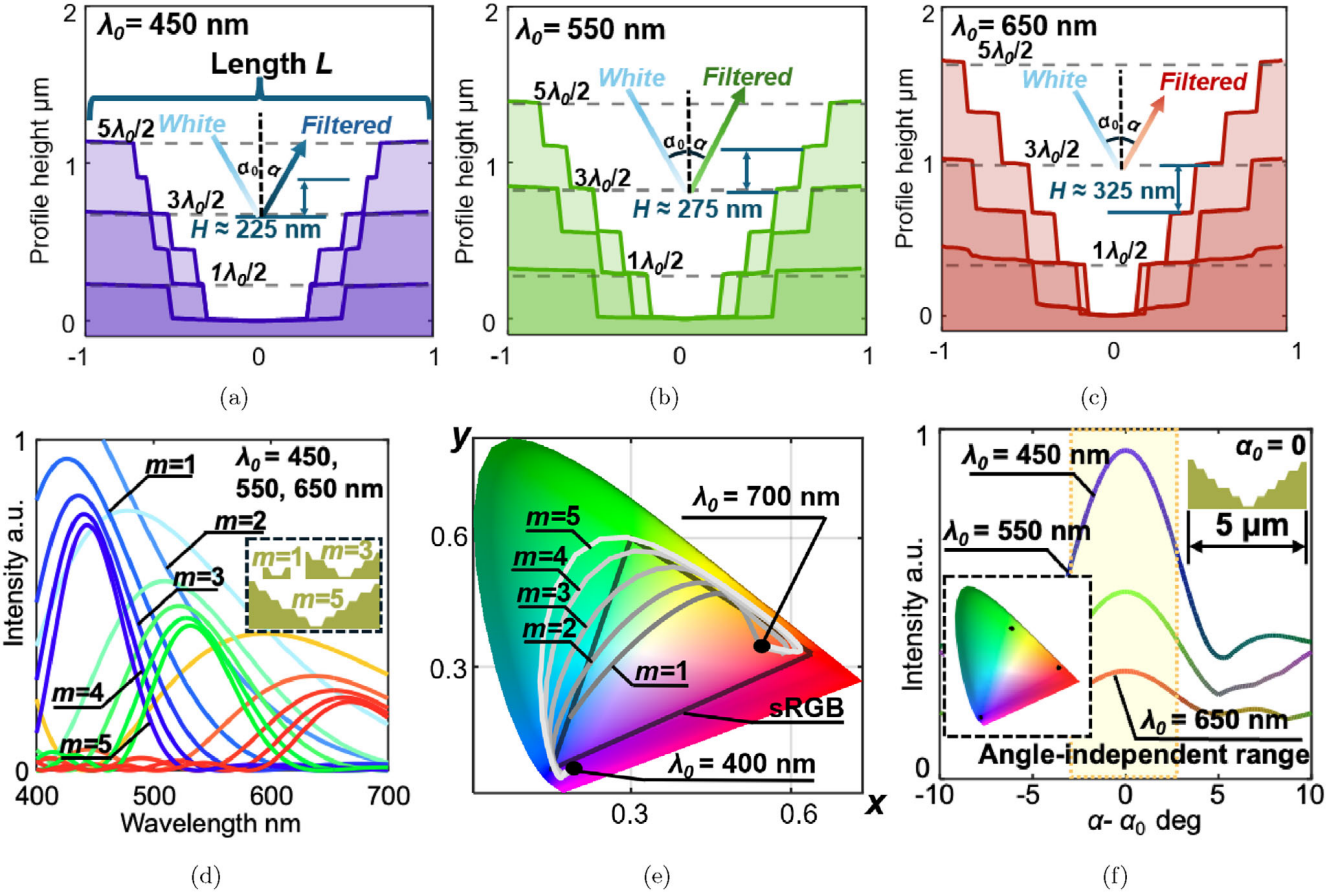
Surface profiles of wavelength‐selective reflectors obtained by global optimization when λ_0_ equals a) 450 nm, b) 550 nm, c) 650 nm; d) reduction of the spectrum bandwidth as the number of staircase levels, *m*, increases from 1 to 5; e) color gamut of the designed surface when the number of staircase level *m* changes from 1 to 5; f) the simulated color changing with observation angle α when the reflector length *L* = 5 μ m.

The wavelength‐selective reflector requires a deliberately designed surface profile to produce locally angle‐independent color with specified wavelength only by a surficial structure. In our approach, we formulated a specialized optimization problem and employed a genetic algorithm for global search. The resulting surface profiles and corresponding spectra for three exemplary wavelengths λ = 450, 550, 650 nm are plotted in **Figure** **2**, respectively. The x‐axis in these figures is normalized from ‐1 to 1. From the surface profiles displayed in Figure 2, we observe that the resulting surfaces tend to converge to a staircase‐like profile when globally optimized. Specifically, the step height *H* of the staircase is approximately half of the target wavelength λ_0_. For cases where the incident angle α_0_ ≠ 0, we extend this conclusion to a more generalized form, accounting for variations in incident angles:

(1) $$\begin{array}{c} \lambda_{0}=2H\cos\alpha_{0} \end{array}$$

where the peak wavelength of the produced color λ_0_ is related to the step height *H* and the incident angle α_0_. The reflected wavelength is determined by the step height of the structure, while the purity and gamut of the color are influenced by the number of levels in the design. As the number of staircase levels increases, the bandwidth of the reflected spectrum narrows and becomes concentrated around the target wavelength shown in Figure 2 (intensity in arbitrary units), resulting in a more defined and preferred color. Based on these characteristics, the optimal surface profile can be described as a “surficial Bragg reflector,” where incident light is filtered by steps of equal increment rather than layers with varying refractive indices. Additionally, increasing the number of levels results in a narrower bandwidth. More importantly, the simplified multi‐level design enhances manufacturability. Unlike other surficial coloration methods, the proposed reflector generates colors independently, without relying on periodic structures. Each segment of the structure produces color individually, further distinguishing it from other approaches.

To validate the feasibility of the surficial wavelength‐selective reflector across the entire visible light spectrum, we calculated the spectra of surfaces composed of steps with a height of λ_0_/2 and equal width. The wavelength λ_0_ was varied from 400 to 700 nm in increments of 10 nm, while the number of levels, *m*, ranged from 1 to 5. The resultant colors were converted into the XYZ color space using the CIE 1931 standard under ideal illumination conditions. The gamut produced by these surfaces is plotted in Figure 2, where the area partially enclosed by the curve represents the achievable color range through color mixing. The curve itself shows the numerically calculated color transitions from blue to red as λ_0_ increases and indicates the limits of the color gamut of given *m*. As *m* increases from 1 to 5, the color gamut expands, indicating enhanced color production capability. When *m* = 5, the color gamut encompasses the entire sRGB space, suggesting that full‐color reproduction comparable to standard displays or cameras is theoretically achievable. This expansion occurs because increasing *m* narrows the bandwidth of the spectrum, as shown in Figure 2. Since *m* = 5 adequately represents the full‐color display by covering the entire sRGB space, we further investigate the angle‐independent effect under this condition.

The angle‐independent range of the surficial wavelength‐selective reflector is determined by the angular separation between the 0th‐order diffraction and the ±1st‐order diffraction. For a given wavelength, a smaller structure length *L* results in a wider angle‐independent range. To balance fabrication feasibility, we set the length of the reflector to 5 μ m, with the smallest features within the structure ranging from 200 to 300 nm, which approaches the limits of diamond machining capabilities. The simulated color and intensity observed from different viewing angles for a single reflector are shown in Figure 2. Around the 0th‐order diffraction, a consistent color display is maintained, while the intensity decreases with increasing observation angle. Within this angle‐independent range, the color variation converges to single points in the color gamut (as depicted in Figure 2), confirming consistent color display across the observed angles.

### 2.2 Surficial Wavelength‐Selective Reflectors Fabricated by Modulation‐Assisted Machining

**Figure 3:**
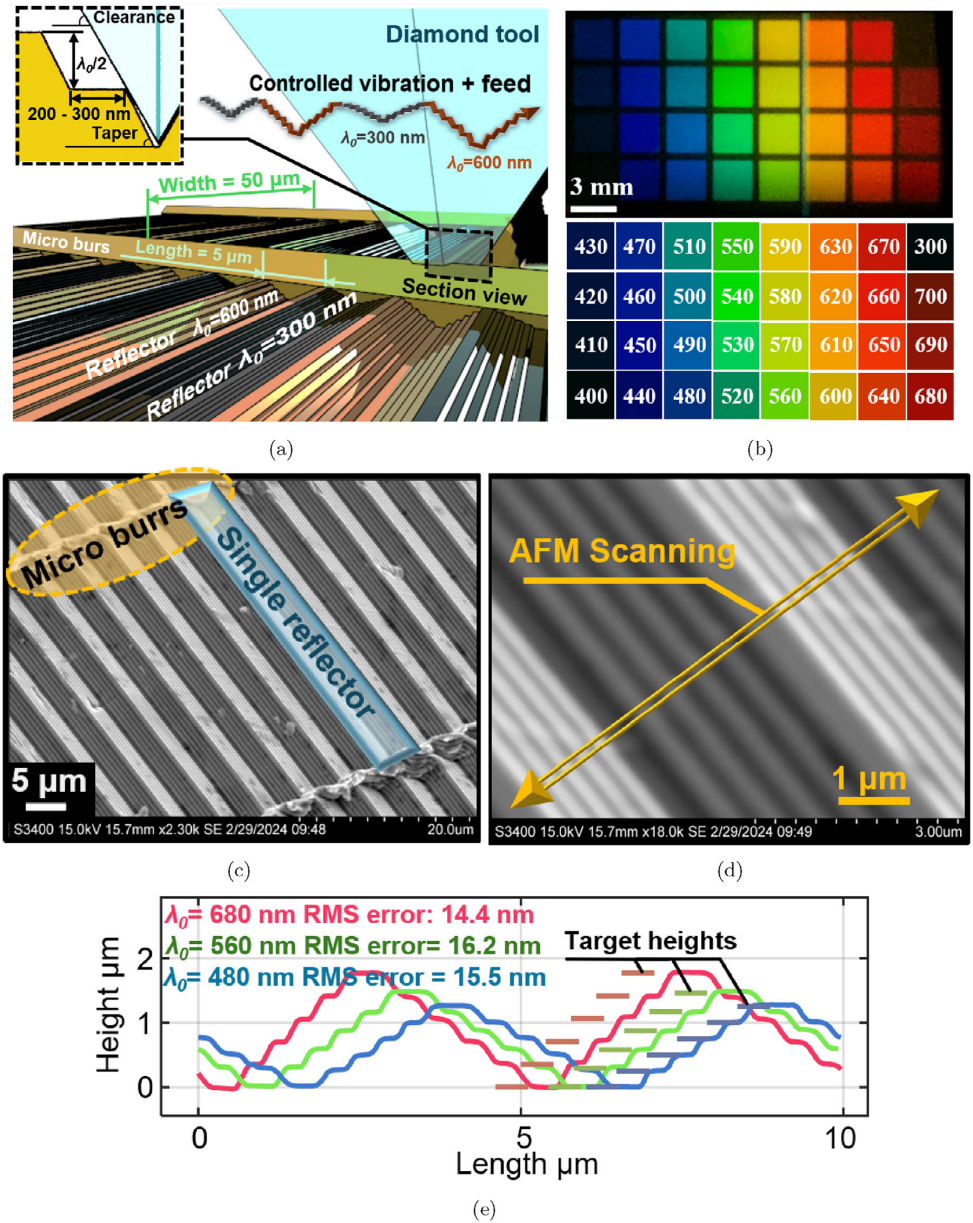
a) Reflectors of different λ_0_ fabricated by modulation‐assisted UPM; b) fabricated color blocks with changing λ_0_ captured at α = 15° and the table of corresponding targeting wavelengths; c) 2300 × and d) 18000 × SEM images of machined surficial wavelength‐selective reflector; e) surface profile of fabricated wavelength‐selective reflectors measured by AFM.

Nanometer‐level forming errors in the structure can significantly affect the resulting color because variations on the order of tens of nanometers in wavelength can cause significant changes in the perceived color.^[^ ^46^ ^]^ To address this, we propose the use of modulation‐assisted machining, where a nanometric structure is directly machined by a single‐crystal diamond tool following a precise trajectory, as illustrated in **Figure** **3**, to ensure high forming accuracy and surface quality. In our demonstrated process, the length of the reflector, which is along the tool lateral feed direction, is set to 5 μ m due to the balance between angle‐independence range and manufacturability. Then, a taper angle is applied between levels to avoid the flank face interference (taper angle < clearance angle), and the length of each level of the reflector can be down to 200 to 300 nm. The width of the reflectors is 50 μ m, which matches the edge width of the diamond tool. By using well‐calibrated tool modulation to cut reflectors in sequence, the adjacent reflectors can have different target wavelength λ_0_ (300 and 600 nm are used as examples in Figure 3), to produce variable colors in a pixelated format that periodic structure designs are not capable of. The coverage speed of the demonstrated process is approximately 2 mm^2^min^−1^ at a modulation frequency of 125 Hz.

Using this deliberately designed modulation‐assisted machining method, we successfully fabricated a series of color blocks with target wavelengths (λ_0_) ranging from 400 to 700 nm, increasing in 10 nm increments, on the surface of 360 Brass. To enhance visibility, the incident angle was set to 15° to separate the incident and reflected light. Additionally, a block with λ_0_ = 300 nm, in the ultraviolet range, was added to demonstrate the appearance of “black.” As shown in Figure 3 captured by a camera located at α = 15°, the color gradually changes from deep blue to deep red, following the expected sequence illustrated in Figure 3. It is noteworthy that the background of the machined area in Figure 3 is also fabricated with λ_0_ = 300 nm, in addition to the upper‐right block. This demonstrates that an applicable black background is achieved on an illuminated brass surface, which is generally considered to be reflective.

SEM images were captured at random locations on the color blocks to examine the fabrication quality of the surficial wavelength‐selective reflector. The 2300 × magnification image (Figure 3) shows the reflectors with each individual dimension of 5 μ m in length and 50 μ m in width. In the width direction, the reflectors are separated by micro‐burrs caused by the machining process. In the higher magnification image (18000 ×, Figure 3), the fabricated structure clearly displays the intended five‐level (*m* = 5) staircase design with a clean surface finish. To further verify the surface profile, AFM measurements were taken along the direction of the steps, as shown in Figure 3. Color blocks with target wavelengths of λ_0_ = 480, 560, and 680 nm were selected, with their measured surface profiles shown in Figure 3. These profiles confirm the desired five‐level staircase structure, with step heights increasing in proportion to λ_0_. The RMS forming error of the functional range (the flat range) is measured to be from 14.4 to 16.2 nm, indicating that the process can scalably achieve these staircase structures of accuracy better than 20 nm.

### 2.3 Wavelength‐Selective Performance of the Surficial Reflectors

**Figure 4:**
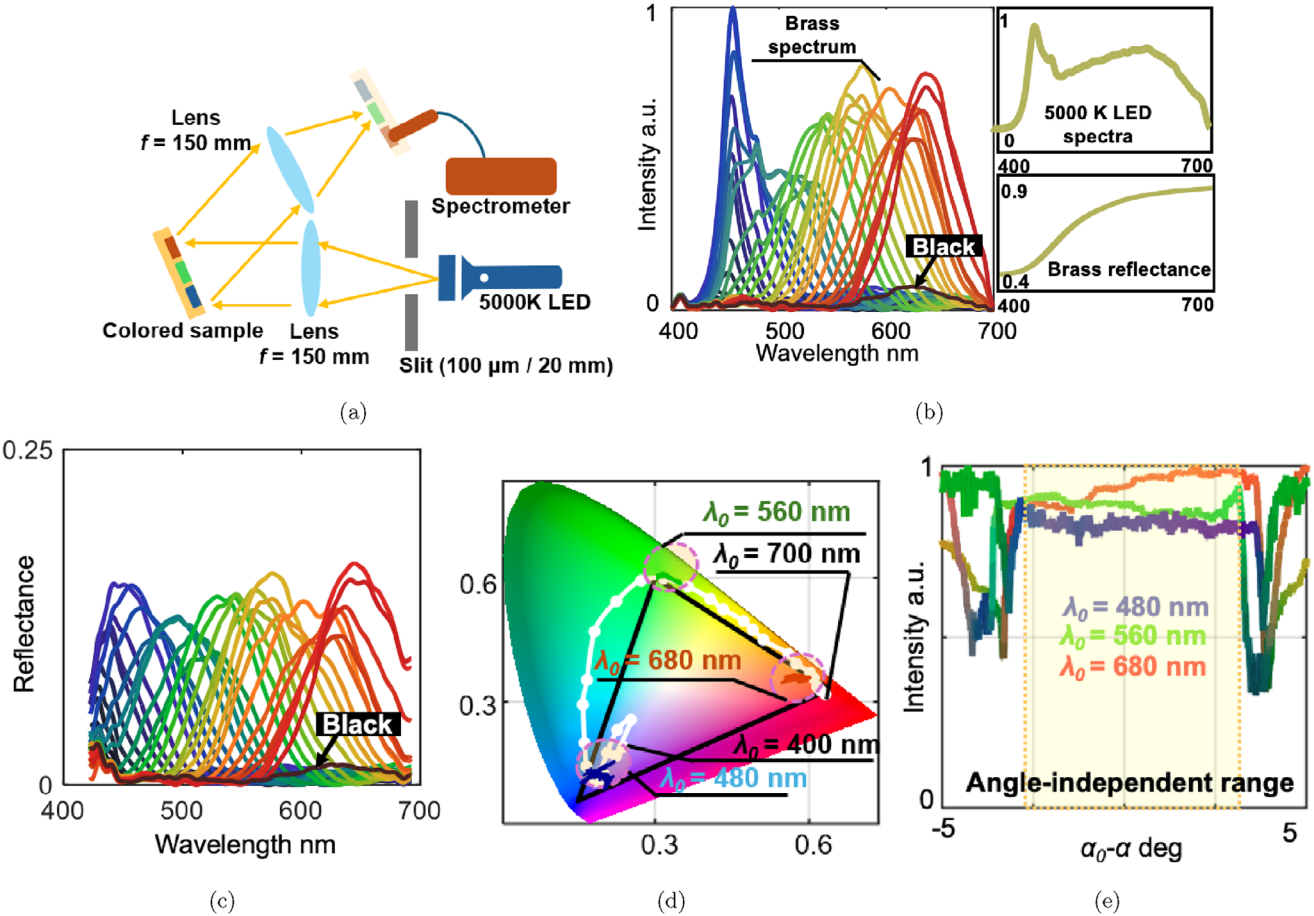
a) An optical system for spectrum measurement; b) measured spectrum of the color blocks; c) the calibrated reflectance spectrum of the color blocks; d) color gamut of the machined color blocks; e) captured color when changing α under non‐paraxial illumination.

To further validate the optical performance of the machined surficial wavelength‐selective reflector, an optical system (shown in **Figure** **4**) was constructed. A 5000K LED was selected as the light source due to its relatively uniform spectral output. The light reflected from the illuminated colored surface was captured by a spectrometer (CCS200, Thorlabs), and the spectra of each color block were recorded. The measured spectra of all 32 color blocks are presented in Figure 4, captured under consistent illumination conditions (100 μ m slit width) and identical spectrometer gain settings. So, all spectral measurements were conducted under identical illumination conditions, and the resulting intensity values were normalized to the maximum within the dataset. As a relative comparison across different target wavelengths, all intensities are reported in arbitrary units (a.u.). The spectral peaks shifted from 450 to 650 nm as the color changed from blue to red. The spectrum for λ_0_ = 300 nm showed very low intensity (globally below 0.1), indicating a suitable “black” presentation, as the ultraviolet light selected when λ_0_ = 300 nm is neither visible in the color space used nor emitted by the light source. It is important to note that although color intensity in theory decreases with increasing wavelength as shown in Figure 2, the reflective properties of brass (measured using the same method on a brass mirror surface) caused the reflectivity at larger wavelengths to be higher. As a result, the peak intensity did not attenuate dramatically as the wavelength increased, as shown in Figure 4. To obtain the reflectance of the wavelength‐selective reflector, the normalized intensity was calibrated using a reference mirror (PF10‐03‐G01, Thorlabs) with known reflectance. The same light source and optical path were used to ensure consistent illumination conditions. The resulting reflectance spectrum is shown in Figure 4. The bandwidth is cropped to 420–680 nm to remove unreliable reflectance calibration results caused by measurement noise at the bandwidth limit. The reflectance remains nearly constant at around 15% across the measured wavelength range. This also indicates the balance between two competing effects: the decreasing light intensity at longer wavelengths and the intrinsic reflectance of the brass substrate.

The colors of each block are plotted on the chromaticity diagram in Figure 4, where the gamut covers nearly the entire sRGB space, indicating the potential for full‐color presentation. Thus, we can define our RGB color directly using colors demonstrated in Figure 3 to achieve full‐color performance comparable to the sRGB standard.

To establish an RGB color base, the color blocks with λ_0_ = 480, 560, and 680 nm were selected as the three base colors. To validate the angle‐independent effect, these three color blocks were illuminated through a 20 mm square aperture instead of a slit, allowing for variations in incident angles within a range of 7.6°. The spectra were captured at different observation angles α, within a range of |α − α_0_| < 5°, with increments of 0.014°. The spectra were converted to their corresponding colors using the CIE 1931 standard and plotted in Figure 4, showing brightness changes. Over a range greater than 5°, the results demonstrate nearly consistent color and grayscale values. The XYZ coordinates of the colors within this range were plotted in Figure 4 and converged to three distinct points, confirming the local angle‐independent effect. To be noted, this test shows the maximum angle‐independent range achievable with the current structure design, where *L* = 5 μ m. To further increase the angle‐independent range, practically up to 30°, *L* would need to be reduced, though this would come at the expense of brightness.

### 2.4 Full‐Color Presentation of Structural Colored Images

**Figure 5:**
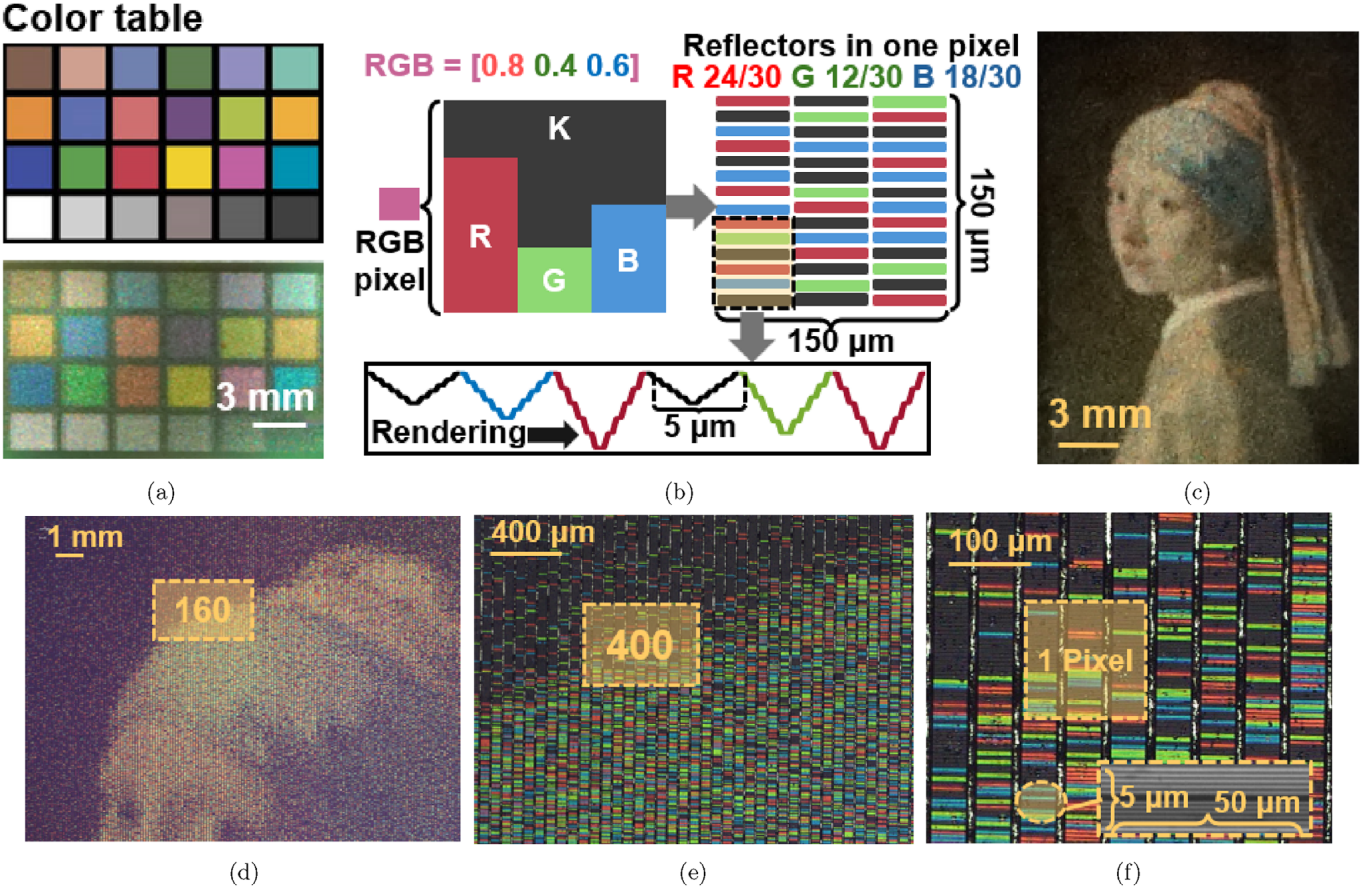
a) Color table (ColorChecker Classic chart) and the corresponding fabricated colored samples on a metallic surface; b) pixel arrangement for RGB‐based color reproduction; c) Girl with a Pearl Earring rendered by the proposed method; microscopic images of the rendered image in increasing magnification (d) 20 ×, e)160 ×, and f)400 ×, respectively).

The color gamut shown in Figure 4 envelops the color space that can be potentially reached by the proposed structure. To generate a specific color within this gamut, such as those represented by the ColorChecker Classic test chart in **Figure** **5**, we choose the RGB color model to mix three base colors with different intensities to reproduce a wide range of colors, including grayscale tones. By referring to the results of color measurements in Figure 4, the reflectors where λ_0_ = 480, 560, 680 nm are chosen to represent blue, green, and red, respectively. In each rendered pixel, the RGB value is reproduced by the number of corresponding RGB reflectors within.

In the rendering process (Figure 5), each surficial wavelength‐selective reflector is assigned dimensions of 50 by 5 μ m, and each pixel measures 150 μ m in both width and length, equating to a resolution of 171 pixels per inch. Consequently, each pixel contains 150 reflectors, with the RGB color values represented by three integers from 0 to 50 in three base colors. For example, to render a single pixel of “purple” with the color code [0.8~0.4~0.6], the pixel would be filled with 40 red, 20 green, and 30 blue reflectors, with the remaining areas filled by black reflectors. The reflectors are then rearranged in either a random or user‐defined sequence within a pixel, allowing the same image to be presented with different pixel arrangements. This flexibility offers the potential for coding hidden information beyond the visible image. For demonstration purposes, the reflectors were arranged randomly, and the reflectors were fabricated in sequence with the sequential profile corresponding to each color reflector, as shown in Figure 5.

Using this pixel rendering strategy, the color table in Figure 5 was successfully rendered on a metallic surface, showing consistent color reproduction compared to the original table. Additionally, the image of Girl with a Pearl Earring was rendered using the same method, as shown in Figure 5. The rendering process of the whole image takes 5 h and 20 min, but efficiency could be significantly improved by increasing the modulation base frequency (currently at 125 Hz). When illuminated by a standard microscope light source and observed under varying magnifications, the hierarchical details of the image, produced by the surficial wavelength‐selective reflectors, are revealed progressively. Zoomed‐in images at different magnifications are displayed in Figure 5. At 20 ×, the enlarged original image is visible, while the RGBK‐based reflector arrangement is revealed at 160 ×. At 400 ×, individual reflectors can be observed, showing their RGB colors or appearing black. Each reflector contains the diamond‐machined nanostructures depicted in Figure 3.

In conclusion, we solve the “impossible trinity” of structure coloration and fulfill the gap between one‐layer structure design and optimal color performance by designing and fabricating a surficial wavelength‐selective reflector. The images rendered by the method include hierarchical structures from millimeter to nanometer scales. The hierarchical structure is machined in a single step by the proposed modulation‐assisted UPM process with nanometer‐level depth accuracy. Different from single‐layer periodic structure design, each surficial wavelength‐selective reflector produces an individual pre‐assigned color, which enables user‐defined coding in pixel arrangement. The hierarchical structure design and coding capability provide a unique strength for potential applications of anti‐counterfeiting and data storage.

## 3 Discussion

In this study, we introduce a novel single‐layer wavelength‐selective reflector capable of producing a broad color gamut with local angle independence, fabricated using a modulation‐assisted ultra‐precision machining process. This method brings scalability and large‐area fabrication within reach, making it suitable for potential industrial adoption. While our current design offers a practical solution to the “impossible trinity” challenge, it also involves certain trade‐offs. These limitations suggest potential directions for future improvements, including the following aspects:

The demonstration of the introduced structures was carried out with careful consideration of the trade‐offs between fabrication efficiency and spatial resolution, as well as machinability and angular independence. The fabrication efficiency is primarily limited by the base driving frequency (125 Hz), which can be increased by several orders of magnitude by employing a vibration tool with a higher bandwidth (such as a commercially available fast tool servo that can run up to 10 kHz, which is about 100 times faster than the demonstrated example). Alternatively, efficiency can be improved by using a tool with a wider cutting edge (currently 50 μ m), at the expense of spatial resolution.

The angular independence of the resulting structure is governed by its period. To achieve high‐performance color rendering, we selected a five‐level structure, yielding a reflector period of 5 μ m, which corresponds to the minimum feasible step width of 200–300 nm. According to the grating equation, the resulting angular spread is approximately 5°, consistent with our experimental validation. If a reduced color gamut is acceptable, the angular independence can be extended up to 30° by fabricating structures with a smaller period of approximately 1 μ m.

The resultant structural color is also influenced by the choice of material and illumination source. In this study, we conducted demonstrations using a 5000K LED light source and a brass substrate. Due to the spectral decay of the reflectors at longer wavelengths and the wavelength‐dependent reflectivity of brass, the resulting measured spectrum reflects these combined effects. To achieve the tailored wavelength‐selective performance, both the material and the illumination source must be selected deliberately. Alternatively, adjusting the area occupied by each type of reflector, particularly those corresponding to high‐efficiency bandwidths (e.g., the blue range shown in Figure 4), can serve as a compensation strategy to maintain a consistent color tone.

Additionally, for further discussions on optical design strategies, modulation‐assisted fabrication techniques, additional characteristics of the wavelength‐selective reflectors, potential mass production feasibility, and long‐term stability, please refer to the Supporting Information.

## 4 Experimental Section

### Global Optimization for Wavelength‐Selective Reflector Design

**Figure 6:**
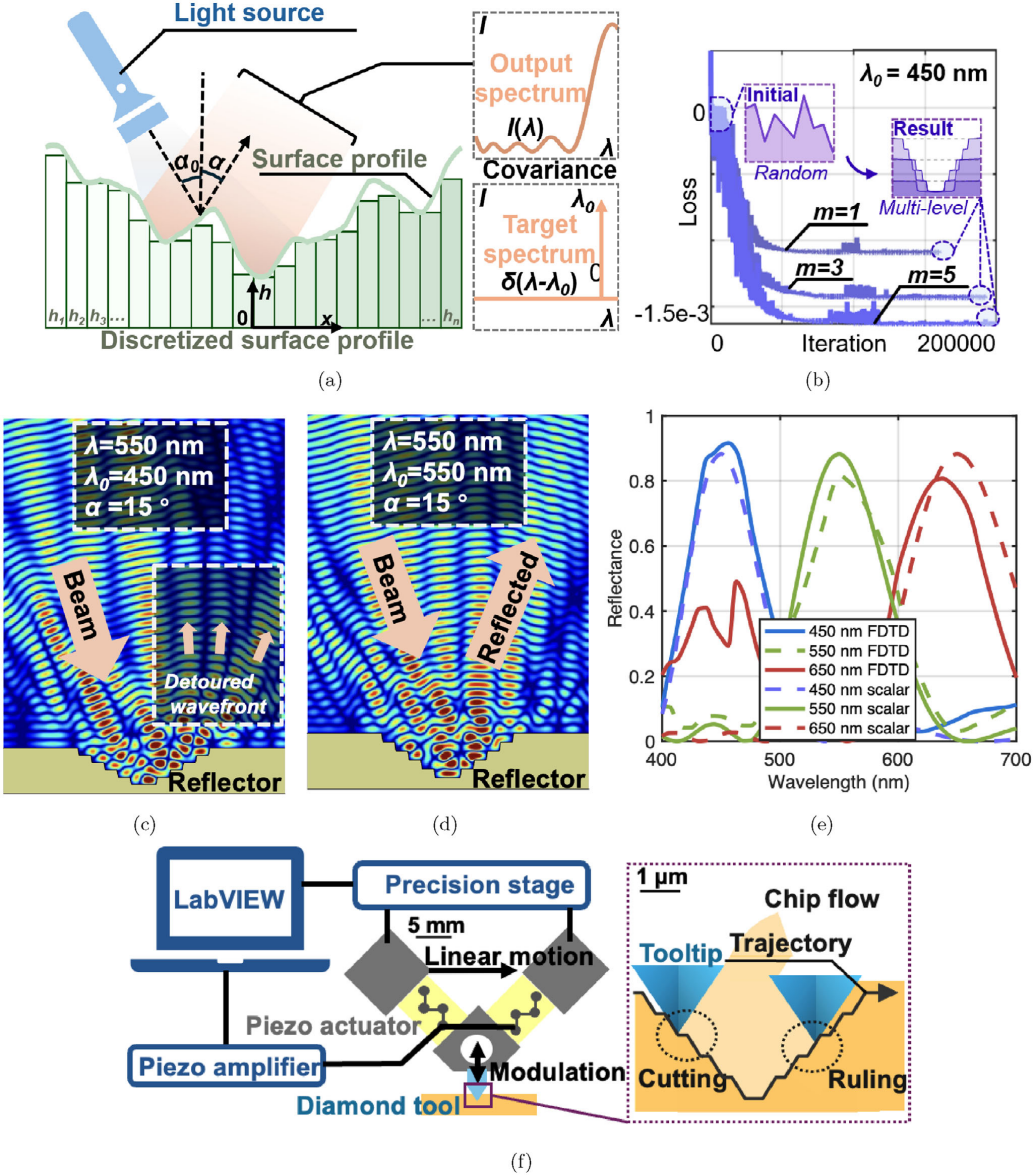
a) Optimization scheme for the design of wavelength‐selective reflectors; b) convergence of genetic algorithm optimization; FDTD simulation of the light propagation above the reflector corresponding to c) λ_0_ = 550 nm and d) 450 nm; e) the comparison between resultant spectra from FDTD and scalar‐based simulation; f) fabrication strategy of the surficial wavelength‐selective reflector.

To identify the optimal surficial structure that achieves wavelength‐selective effects for a given wavelength λ, we discretize the continuous surface into *n* segments with varying heights *h*_*n*_, as illustrated in **Figure** **6**. If the desired color corresponds to a target wavelength λ_0_, the target spectrum can be represented by δ(λ − λ_0_), where δ is the Dirac function. The spectrum produced by the designed surface could be quantitatively simulated using scalar diffraction theory.^[^ ^35^, ^47^ ^]^ The similarity between the produced color and the target color could be evaluated by calculating the covariance of the two spectra. Thus, the search for the optimal surface profile became a process of maximizing the spectrum covariance between the produced and target colors. Based on this principle, the optimization problem can be formulated as follows:

(2) $$\begin{array}{c} \begin{array}{c} \min_{h_{i}}\;-\text{Cov}\;\left(\frac{1}{\lambda^{2}}{\left|\sum_{i=1}^{n}e^{-4\pi j\frac{h_{i}}{\lambda }}\right|}^{2},\delta \left(\lambda -\lambda_{0}\right)\right)\\ \text{subject to}\;\;0\leq h_{i}\leq \frac{m}{2}\lambda_{0},h_{i-1}\leq h_{i} \end{array} \end{array}$$

where *m* is a positive integer, and the upper boundary of *h*_*i*_ is limited by *m*λ_0_/2. In the formulation of the optimization problem, the cost function depends solely on the values of each *h*_*i*_ and is independent of their sequence. To accommodate fabrication constraints, the sequence of *h*_*i*_ values is restricted to be monotonic. The convergence curve of the optimization of λ_0_ = 450 nm is shown in Figure 6. For intuitive visualization, the surface profiles were mirrored symmetrically along the x‐axis, as presented in Figure 2. Additional technical details of the optimization process are provided in the Supporting Information.

Since the scalar‐based theory was an approximation that neglects multiple reflections within the structure while offering high computational efficiency, finite‐difference time‐domain (FDTD) simulations were performed as an additional validation. Representative results of the FDTD simulations are presented in Figure 6. Figure 6 illustrates the light propagation at a wavelength of 550 nm for target wavelengths of 450 and 550 nm, respectively. When the target wavelength is 450 nm, the 550 nm light is detoured to multiple diffraction paths, whereas for the 550 nm target wavelength, the light is reflected with a well‐collimated wavefront, demonstrating the wavelength‐selective reflection mechanism. The reflectance spectra of visible light under ideal conditions (perfectly reflective material with no manufacturing errors) were simulated using FDTD at target wavelengths of 450, 550, and 650 nm and compared with the results from the scalar diffraction theory. The two methods show good agreement in both the spectral distribution and the peak wavelengths across all three target wavelengths, with minor discrepancies observed in the 400–500 nm range for the 650 nm case. Additional details of the FDTD simulations are provided in the Supporting Information.

Although the FDTD and scalar diffraction results exhibit consistent trends, it was noteworthy that the experimentally measured reflectance (around 0.15) is significantly lower than the simulated value (approximately 0.8) or even the considering material reflectance (approximately 0.5). This reduction was likely attributed to the surface integrity of the machined brass and unavoidable fabrication imperfections. Consequently, a more powerful light source was required in practical implementations to achieve the same illuminance as predicted by simulations while preserving the designed color tone.

### Fabrication of Surficial Wavelength‐Selective Filters

The surficial wavelength‐selective reflectors were fabricated using a custom‐designed diamond insert (manufactured by Chardon Tool), which was mounted on a customized piezo tool according to the system configuration shown in Figure 6. The customized piezo tool was mounted on an ultra‐precision lathe with three linear axes and one rotational axis *XYZC* (Nanoform X, AMETEK Precitech). The ultra‐precision lathe performed a constant lateral feed while the piezo tool generated the pre‐calibrated modulation trajectory to drive the diamond tool to fabricate the reflectors on the workpiece surface.

The diamond insert has a flat tip of 50 μ m to machine reflectors of the same width. The rake angle was ‐30° and the clearance angle was 60 ° to avoid interference and achieve a higher aspect ratio, as illustrated on the right‐hand side of Figure 6.

To generate tool modulation following the desired trajectory, a LabVIEW program was developed to produce the driving signal with a sampling frequency of 1 MHz. The signal was amplified by two separate piezo amplifiers (PX200, PiezoDrive), which then drove two piezo actuators (PK44LA2P2, Thorlabs) mounted on the customized piezo tool. This setup ensured that the diamond insert at the tip followed the designed trajectory to form the wavelength‐selective reflectors. A laser vibrometer (CLV‐2534, Polytec) was used to capture the actual trajectory of the diamond insert driven by the customized piezo tool. The recorded trajectory was compared with the desired one, and any errors due to hysteresis were compensated by adjusting the driving signal in LabVIEW. This compensation process was repeated several times until the desired trajectory was achieved, with an error margin of less than 20 nm. After the compensation had been finished, the workpiece was mounted on the ultra‐precision lathe, and the machining process started.

The workpiece material was chosen to be 360 Brass, considering the excellent machinability. The ultra‐precision lathe held the workpiece while driving the piezo tool to move in 3D space and machine the workpiece surface following a raster‐scanning path. The cutting velocity was kept constant at 37.5 mmmin^−1^ to achieve 5 μ m pitch under a driving signal of 125 Hz base frequency.

### Measurement of Fabricated Structures and Rendered Images

The overviews of rendered color blocks and images were captured by a digital camera (α7 paired with SEL90M28G Lens, Sony). The images of fabricated structures with feature sizes in millimeters and micrometers were captured by an optical microscope (VHX‐7000, Keyence). The SEM images showing nanometer‐level features were captured by a scanning electron microscope (S‐3400N‐II SEM, Hitachi), and the nanometric surface profile was measured by an AFM (Dimension Icon, Bruker). The spectrum of the produced color was measured by a spectrometer (CCS200, Thorlabs). The fiber head was located on a rotary stage to capture the spectrum from different perspectives.

## Conflict of Interest

The authors declare no conflict of interest.

## Supporting information

Supporting Information

## Data Availability

The data that support the findings of this study are available from the corresponding author upon reasonable request.
