# Supplementary material for: Nanometric Surficial Reflectors: Achieving High‐Performance and High‐Throughput Structural Coloration Through Modulation‐Assisted Machining
Source: Adv Sci (Weinh). 2025 Nov 5;13(4):e06162. doi: 10.1002/advs.202506162 (PMC12822476; doi:10.1002/advs.202506162)
Supplement: Supplementary file 1 — Supporting Information [file ADVS-13-e06162-s001.pdf]

Supplementary material:

Nanometric Surficial Reflectors: Achieving High-Performance and High-Throughput Structural Coloration through Modulation-Assisted Machining

Yaoke Wang[1], Malachi Landis[1], and Ping Guo[1,*]

[1]Northwestern University, Department of Mechanical Engineering, Evanston, IL, 60208, USA
[*]ping.guo@northwestern.edu

1 Optical Design

1.1 Optical design of wavelength-selective reflectors using genetic algorithm

To determine the optimal optical surface fulfilling all the desired features: local angle independence, full-color feasibility, and surficial structure design, an optimization-based method is applied to search for the optimal optical surface structures. Due to the prerequisite of a surficial structure, wavefront interference induced by the fluctuating surface profile dominates color generation. For the convenience of optimization formation, the light propagation is modeled by the scalar-based theory[1,2] in the study. For most of the application scenarios, the distance between the light source and the observer to the structurally colored item is far larger than the scale of the colored area. Here, we assume that the light incidents on the structural colored surface with incident angle α_0 and the observer or detector is at the angle α. Under the paraxial approximation where α_0 and α are small, the resultant wavefront distribution $E(\alpha,\lambda)$ of variable wavelength λ at angle α can be modeled by Fraunhofer diffraction theory:

$$E(\alpha,\lambda) = \frac{A}{\lambda}\int_{-\infty}^{\infty} U(x,\lambda)\, e^{-2\pi j \frac{\alpha}{\lambda} x} dx, \tag{S1}$$

where A is a constant under paraxial assumption when observed from the same distance and $U(x,\lambda)$ is the wavefront distribution on the diffractive surface. When the incident angle α_0 satisfies the paraxial assumption, the $U(x,\lambda)$ can be written by:

$$U(x,\lambda) = e^{-4\pi j \cos(\alpha_0)\frac{h(x)}{\lambda}} e^{2\pi j \frac{\alpha_0}{\lambda}}. \tag{S2}$$

Then, the resultant wavefront can be regarded as composed of two terms. One is the modulation of the diffractive surface, while the other is the propagation term, which is related to the observation angle and incident angle:

$$E(\alpha,\lambda) = \frac{A}{\lambda}\int_{-\infty}^{\infty} \underbrace{e^{-4\pi j \cos(\alpha_0)\frac{h(x)}{\lambda}}}_{\text{Surface}} \underbrace{e^{-2\pi j \frac{\alpha-\alpha_0}{\lambda} x}}_{\text{Propagation}} dx. \tag{S3}$$

According to Eq. S3, as long as the propagation term changes with α, the resulting $E(\alpha,\lambda)$ will change collaboratively. To eliminate the rainbow effect (angle-dependent color) and achieve the local angle-independent effect, we choose to consider the 0th order diffraction only, where $(\alpha-\alpha_0)\simeq 0$. This indicates that the targeting structure color will only show up near the angle of 0th-order diffraction, which can be regarded as a "colored reflection". As a result, the propagation term is approximately 1, indicating a single color only determined by the surface profile $h(x)$ shows up at $\alpha=\alpha_0$. Then, the relative intensity of different wavelengths can be written by:

$$I(\lambda)=\frac{I_s(\lambda)}{\lambda^2}\left|\int_{-\infty}^{\infty} e^{-4\pi j\cos(\alpha_0)\frac{h(x)}{\lambda}}dx\right|^2, \tag{S4}$$

where $I_s(\lambda)$ is the normalized spectrum of the light considering the light source and material reflectivity. Here we assume that the light source has an ideal uniform spectrum and is normally incident on the surface of perfect reflection, where I_s is constantly equal to 1 and $\alpha_0=0$ to simplify the searching process. This indicates that the observed spectrum $I(\lambda)$ is only related to the summation of the phase shift over the diffractive surface $h(x)$.

We discretize the continuous target surface to n segments of different heights h_n and form the optimization problem written by:

$$\begin{gathered}\min_{h_n}\ -\mathrm{Cov}\left(\frac{1}{\lambda^2}\left|\sum_{i=1}^{n} e^{-4\pi j\frac{h_i}{\lambda}}\right|^2, \delta(\lambda-\lambda_0)\right), \\ \text{subject to } \ 0\le h_i\le \frac{m}{2}\lambda, h_{i-1}\le h_i,\end{gathered} \tag{S5}$$

where m is a positive integer and $m\lambda/2$ limits the upper boundary of h.

As shown in Figure S1, the optimization process begins with a randomly discretized surface, represented by a set of lateral positions x_n and corresponding heights h_n. A scalar diffraction simulation is performed to calculate the output spectrum $I(\lambda)$, which is then compared to a target spectral distribution $\delta(\lambda-\lambda_0)$. The covariance between the simulated and target spectra is used as the cost function in a genetic algorithm. If the maximum generation number is not reached, the random surface is iteratively updated under the constraint $h_n < m\lambda_0/2$. Upon convergence, the algorithm outputs the optimized surface profile x_n, h_n.

The built-in genetic algorithm from MATLAB is used to solve the optimization globally. The number of segments is 40, where the number of population is 400 for the genetic algorithm. The convergence curve and corresponding optimization results where $\lambda_0 = 450,\ 550,\ 650$ nm are shown in Figure S2a, S2b and S2c.

The resultant spectrum where $\lambda_0 = 450,\ 550,\ 650$ nm and $\alpha_0=0$ is calculated by Eq. S4 and shown in Figure S3a, S3b and S3c. The corresponding produced color presented on the curve is calculated by CIE 1931[3,4]:

$$\begin{cases} X=\int_\lambda I_s(\lambda)I(\lambda)\bar{x}(\lambda)d\lambda \\ Y=\int_\lambda I_s(\lambda)I(\lambda)\bar{y}(\lambda)d\lambda \\ Z=\int_\lambda I_s(\lambda)I(\lambda)\bar{z}(\lambda)d\lambda, \end{cases} \tag{S6}$$

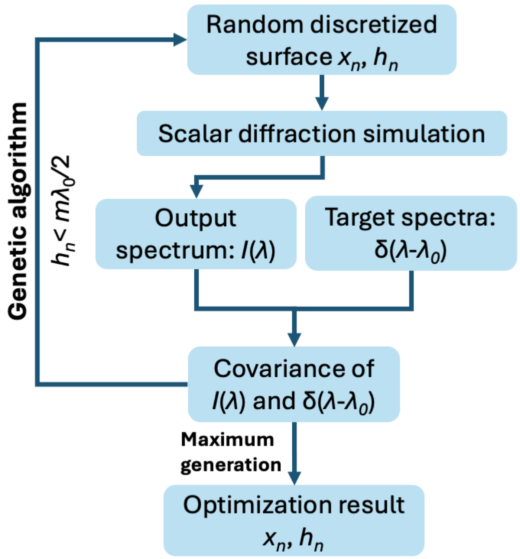

Figure S1. Flowchart of the optimization for wavelength selective effect

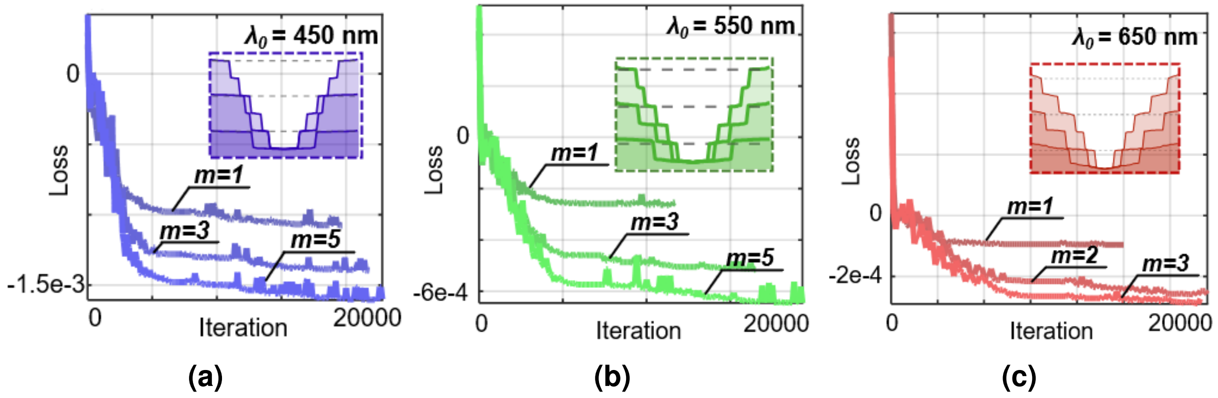

Figure S2. Convergence curve and corresponding optimization result when λ_0 equals a 450 nm, b 550 nm, c 650 nm.

where I_s is the light source spectrum, which is assumed to be 1 for ideal illumination conditions. $\bar{x}, \bar{y}, \bar{z}$ are functions of the chromatic response of the observer from CIE 1931.

The analysis above demonstrates the wavelength-selective performance under the assumption of $\alpha_0 = \alpha = 0$. To further validate the feasibility of a single reflector over changing perspectives, the light intensity of different wavelengths and observation angles are calculated when $m = 5$ and shown in Figure S4a, S4b and S4c.

Simulation results show that maximum intensity occurs when the observation and incident angles match ($\alpha = \alpha_0$) and the observed wavelength equals the target wavelength ($\lambda = \lambda_0$), indicating that the target wavelength λ_0 is emitted under 0th-order diffraction. Here, the 0th-order diffraction can be regarded as a "wavelength-selective reflection." Part of the eliminated light is detoured to the first-order diffraction. Thus, near the 0th-order diffraction range, consistent color is produced, providing a local angle independence.

To demonstrate that the multi-level reflector design can be extended across the entire visible spectrum, structures with different step heights H were simulated. The design wavelength λ_0 was varied from

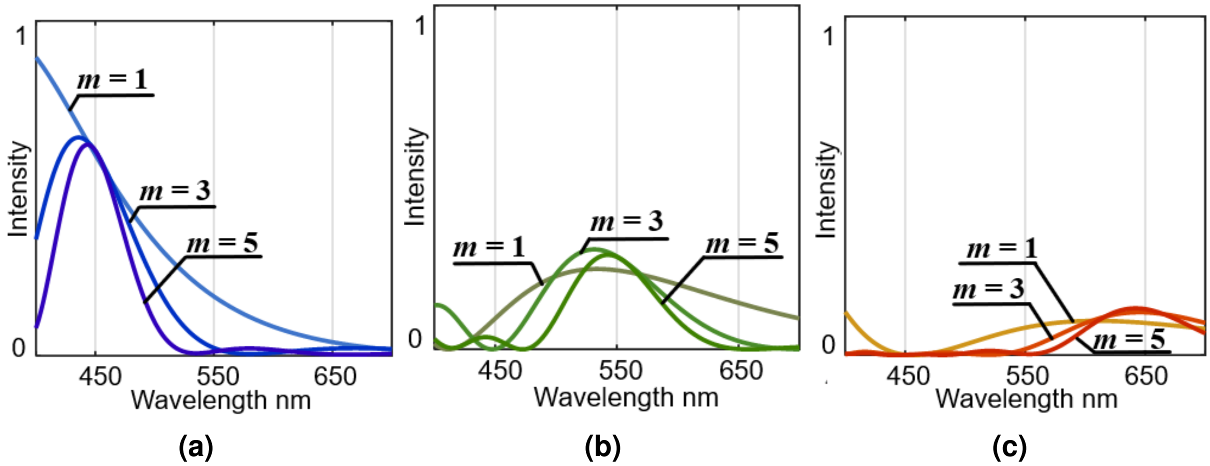

Figure S3. Calculated spectrum induced by the resultant surface profile when λ_0 equals a 450 nm, b 550 nm, c 650 nm.

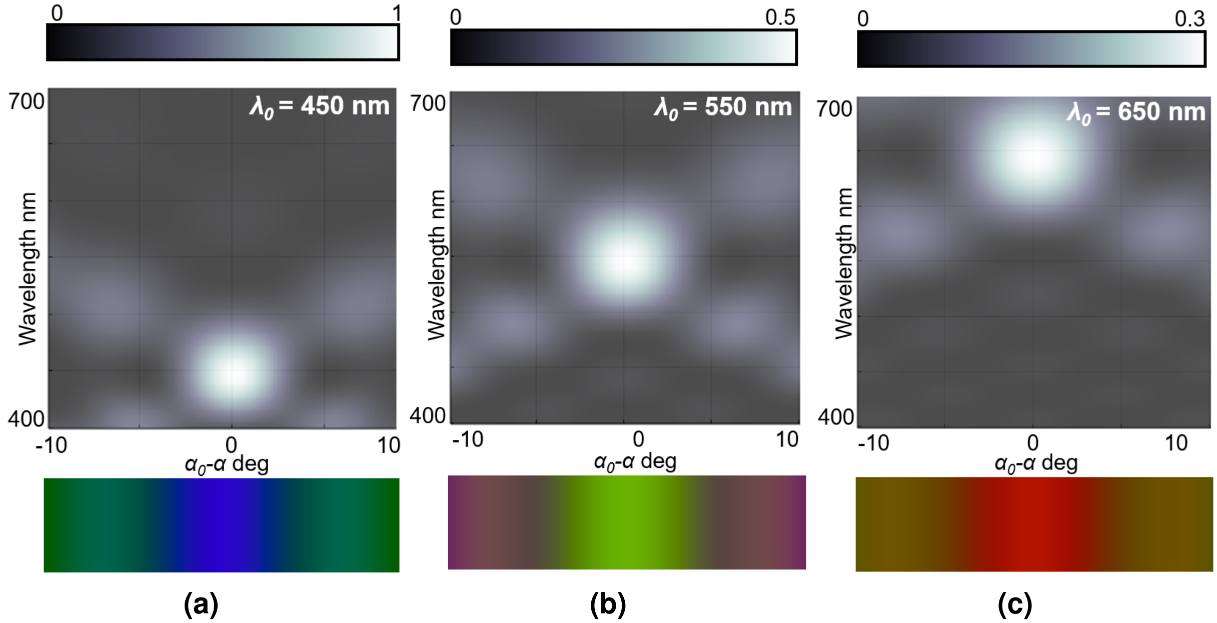

Figure S4. The intensity distribution of different wavelengths and observation angles and corresponding color of changing perspectives when λ_0 equals a 450 nm, b 550 nm, c 650 nm.

400 nm to 700 nm in 10 nm increments, and the corresponding reflectance spectra are summarized in Figure S5. As shown in Figure S5, the peak reflectance shifts systematically from deep blue to deep red as λ_0 increases, with the peak wavelength closely following the designed λ_0. The simulated reflectance values account for the standard reflectance of a brass substrate (typically $\sim$0.3–0.5 in the visible range), ensuring realistic spectral amplitudes.

It is noteworthy that the experimentally measured reflectance (approximately 0.15) is substantially lower than the theoretical reflectance limit of the brass substrate itself (approximately 0.3 to 0.5). This discrepancy is primarily attributed to the finite surface quality of the machined brass reflectors, where micro-scale roughness and unavoidable fabrication imperfections introduce additional optical losses through scattering and absorption.

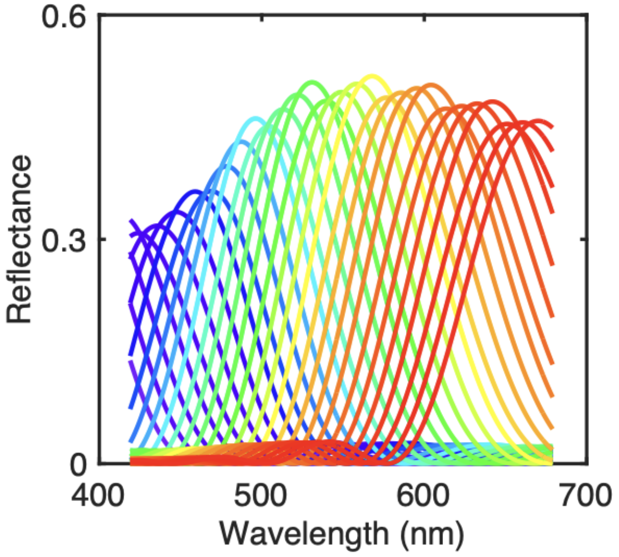

Figure S5. Simulated reflectance spectra with λ_0 ranging from 400 to 700 nm on brass.

1.2 FDTD validation of the wavelength selective reflector

An two-dimensional finite-difference time-domain (FDTD) simulation was developed in MATLAB to validate the propagation and reflection of light waves above the designed wavelength-selective reflector. The computational domain was discretized on a uniform grid with a spatial resolution of $\Delta x = \Delta y =$ 6.25 nm, ensuring numerical stability under the Courant condition with a Courant factor of $S = 1/\sqrt{2}$. A time-harmonic line source illuminated the reflector at an oblique incidence angle of $\alpha = 15°$, with the incident wavelength swept from $\lambda = 400$ nm to 700 nm in increments of 2 nm. Ideal reflection conditions were imposed by setting both the electric and magnetic fields to zero inside the wavelength-selective reflector region.

For each wavelength, the total simulation time was 5600 steps, and the reflected electric field $E_z(x,y,t)$ was recorded over the entire spatial domain.

The results for the 550 nm light reflected by the reflector designed for $\lambda_0 = 550$ nm, shown in Figure S6a, indicate that the reflected wavefront remains nearly symmetric with respect to the incident beam, with most of the optical energy concentrated along the specular direction $\alpha = \alpha_0 = 15°$. In contrast, for reflectors with $\lambda_0 = 450$ nm and $\lambda_0 = 300$ nm, as shown in Figures S6b and S6c, the reflected field becomes distorted, and the optical energy is partially scattered into multiple directions. This scattering effect demonstrates significant attenuation at off-resonant wavelengths, thereby highlighting the wavelength-selective nature of the reflector.

To further quantify the wavelength-selective behavior, the reflectance was calculated by performing a two-dimensional Fourier transform in the region indicated in Figure S7a. The reflected intensity at the observation angle $\alpha = \alpha_0$ was extracted in the spatial-frequency domain and normalized by the corresponding intensity obtained from a reference simulation of an ideal mirror. The reflector geometries were parameterized by their target wavelengths $\lambda_0 = 450$, 550, and 650 nm, and the reflectance spectra were computed across the visible range with a wavelength increment of 2 nm. A comparison of the spectra obtained from FDTD simulations and the scalar diffraction theory is provided in Figure S7b.

The FDTD-simulated reflection spectra exhibit clear peaks at the designed wavelengths, consistent with the scalar theory results. This confirms that the proposed reflector structures maintain spectral selectivity

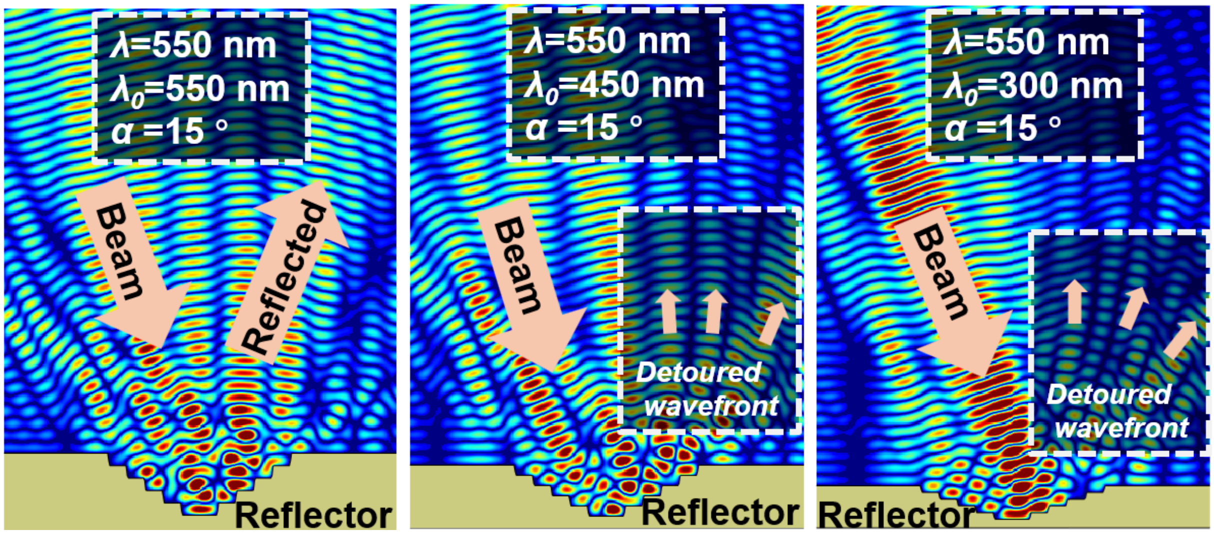

(a) **(b)** **(c)**

Figure S6. FDTD simulation of the light propagation above the reflector corresponding to a λ_0 = 550 nm, b 450 nm and c 300 nm.

Table 1. Key parameters used in the FDTD simulation.

Parameter	Value / Description
Spatial grid size	$\Delta x = \Delta y = 0.00625\ \mu$m
Time step	$\Delta t = S \cdot \Delta x/c$, Courant factor $S = 1/\sqrt{2}$
Domain size	$800\times$ cells per reflector, total 2400×2400
Target wavelengths	400–700 nm, $\Delta\lambda = 6.25$ nm
Simulation time steps	5600 steps per wavelength
Material permittivity	$\varepsilon_0 = 8.854 \times 10^{-12}$ F/m, $\mu_0 = 4\pi \times 10^{-7}$ H/m

across different design wavelengths.Overall, the FDTD simulations confirm that efficient reflection occurs primarily when the reflector step height is commensurate with the incident wavelength, thereby optimizing reflection for the design wavelength while suppressing it at other wavelengths.

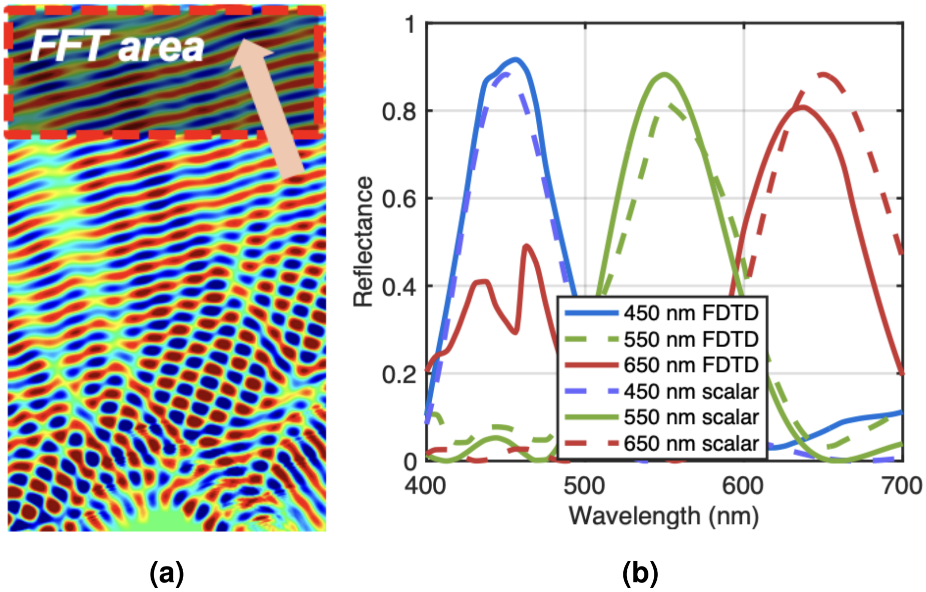

Figure S7. a FFT conducted for calculating the spectra λ_0 = 550 nm at the specific observation angle; b the comparison between resultant spectra from FDTD and scalar-based simulation.

2 Fabrication of surficial wavelength-selective reflectors using modulation-assisted machining

In this study, we propose to use the modulation-assisted machining method to fabricate the surficial wavelength-selective reflector on the metallic surface. The diamond tool will move laterally on the material while vibrating normally to the material with the given modulation trajectory. Similar to the mechanical vinyl etching technique, the time-domain modulation will leave a space-domain micro-structure on the machined surface. The resultant structure of the pre-designed step height will display the corresponding color on the surface. However, achieving nanometer-level accuracy in the depth direction poses significant challenges to the fabrication process. To realize the nanometer-level machining accuracy on a relatively high frequency, we use the piezo tool composed of piezo actuators to drive the modulation of a diamond tool. In the process, Labview will control the precision stage to drive the tool to move laterally while sending the modulation signal in real-time to the piezo amplifier, which will modulate the diamond tool in the micron range with nanometer-level accuracy. During the period of modulation, the tool tip will first cut the material, and remove the material with shear deformation. On the other side of the staircase structure, the tool mainly pushes the material to shear and flow due to a negative equivalent rake angle, which is similar to a ruling process. To avoid tool tip interference with the material, which may cause undesired deformation, we assign that the maximum slope of the structure is restricted to the same as the diamond tool's rake angle and relief angle.

The positioning error of the piezo actuator and the resonant vibration of the precision stage (the machine tool) in the depth direction are the two main factors that challenge the nanometer-level accuracy. The displacement accuracy of the piezo actuator is calibrated to within 10 nm specifically for staircase modulation, measured in real-time using a laser vibrometer with the setup shown in Figure S8a. In the compensation process, the displacement of the cutting tool induced by the input voltage is measured using a laser vibrometer mounted on a vibration-isolated stage. The error between the desired displacement and the measured displacement is computed in LabVIEW through direct comparison. The input voltage is then

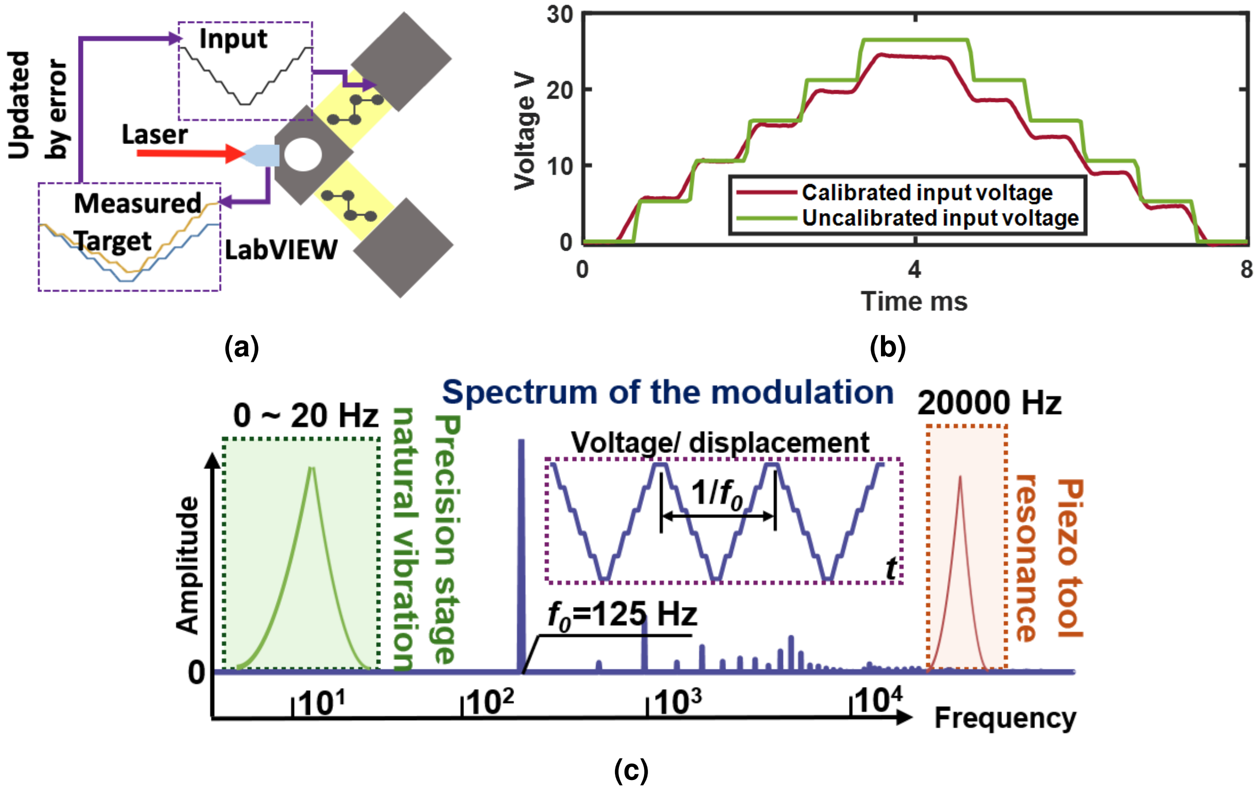

Figure S8. a Calibration framework of the vibraiton cutting tool; b calibrated and uncalibrated input voltage in one period of cutting; c frequency limitation of the modulation.

iteratively updated by adjusting it with the error scaled by a relaxation factor. The compensation process is considered complete when the global displacement error converges within a peak-to-peak amplitude of less than 10 nm.

When the base frequency is set to 125 Hz, the calibrated and uncalibrated input voltage in one period of cutting is shown in Figure S8b. The calibrated voltage input shows an asymmetric path in the increasing and decreasing range due to the existence of hysteresis.

However, due to resonance in the precision stage, disturbances of tens of nanometers in the depth direction cannot be fully eliminated, the frequency bandwidth of which is typically within 20 Hz. To avoid the influence of this disturbance, the solution is to keep the modulation frequency considerably higher than the natural frequency of the precision stage. Then, because the machining process is "faster" than the disturbance, each machined wavelength-selective reflector is locally accurate while the influence of the disturbance is negligible on a larger scale. As shown in Figure S8c, the base frequency of the modulation (f_0), which corresponds to the structure length L, is assigned higher than the bandwidth of the resonant vibration of the precision stage. However, the high-frequency components of the modulation may stimulate the resonant vibration of the piezo tool and cause noisy modulation. Thus, the main parts of the high-frequency components are kept below the resonant frequency of the piezo tool, which is characterized to be 20 kHz. As a result, the available base frequency f_0 is restricted and here we assign it to be 125 Hz. To be noticed, the piezo tool is supposed to respond to the displacement of several kHz before reaching its resonance, even though the base frequency f_0 is only 125 Hz. Thus, a custom-designed piezo tool (the image of the piezo tool is shown in Figure S9a) is applied instead of a commercial fast tool servo system. The piezo tool is equipped with a diamond insert at its tip. A microscopic image with dimensions is shown

in Figure S9b.

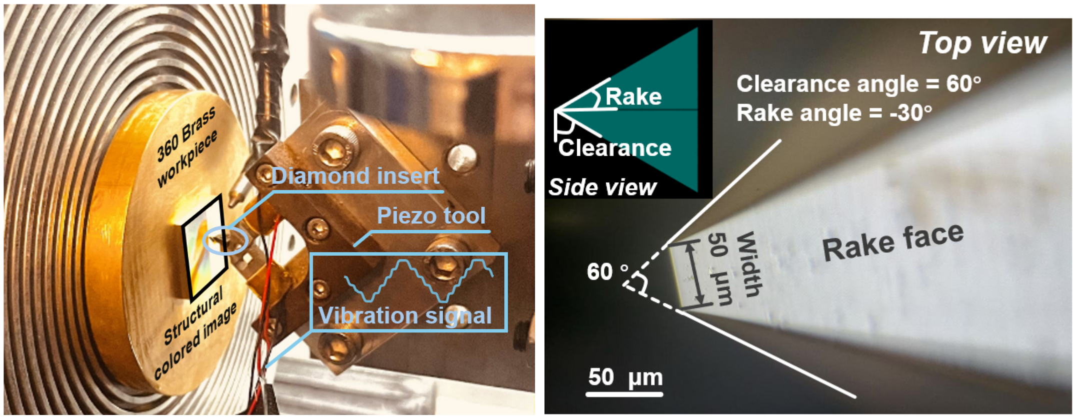

(a) (b)

Figure S9. a A customized piezo tool renders structural colored images on the brass workpiece; b microscopic image (400×) and dimensions of diamond insert.

3 Additional information for the fabricated samples

3.1 Polarization

The validate the influence of polarization of the reflectors, the sample was illuminated with an unpolarized light source, and images were taken using a polarization filter aligned along the S and P directions, as shown in Figure S10a. Slight color differences are visible in the short-wavelength region ($\lambda_0 < 480$ nm) with comparable brightness. Overall, the color transition remains similar between the two cases.

3.2 Hot-stamping test

We fabricated an optical mold with wavelength-selective reflectors on a brass substrate. Replication tests were carried out using a commercial compact hot stamping machine (90DSS, FastToBuy). PMMA was selected as the stamping material. During the process, the mold was heated to 140 °C and the stamping time was set to 10 seconds. The replicated structure was examined under a microscope, and the staircase-like features were successfully reproduced, as shown in Figure S10b. This result demonstrates the basic feasibility of our replication approach. However, achieving full-color image replication will require further optimization of material selection and structural design.

3.3 High-order diffraction

As shown in Figure S10c, we photographed the fabricated color blocks under different diffraction orders (from -2nd to 2nd). On higher-order diffraction, no specific high-saturation color as we presented in the 0th order diffraction is observed. The resulting color appearance is mainly governed by the structural period and the observation angle, with additional influence from the detailed fabricated features. This result highlights the order-selective nature of the structural coloration and emphasizes the effectiveness of zero-order diffraction design in achieving angle-independent and vivid color appearance.

3.4 Long-term protection

Here we provide an approach for the long-term protection of the fabricated nanostructure, as shown in Figure S10d The method involves applying a transparent coating, such as acrylic spray or transparent

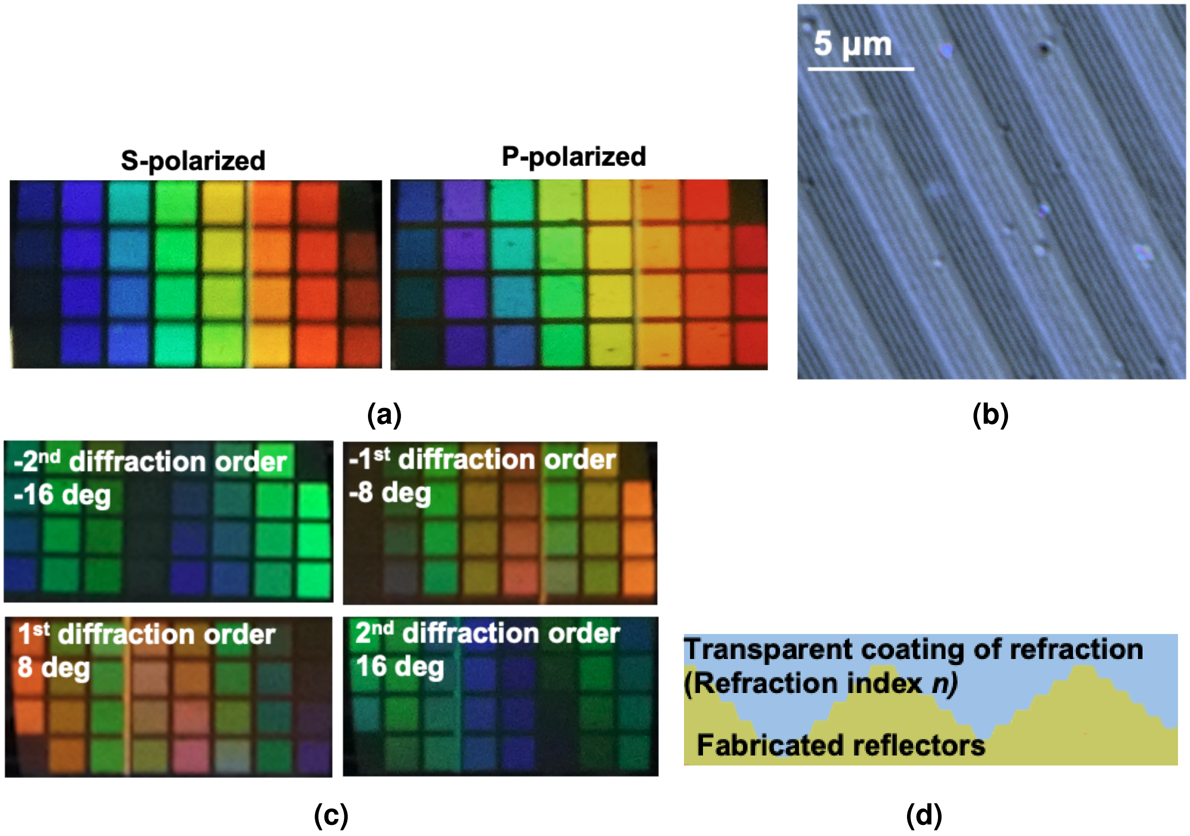

Figure S10. a Comparison of the fabricated color block under s-polarization and p-polarization; b microscopic images of hot-embossed wavelength-selective reflectors; c captured images of fabricated color blocks on high-order diffraction; d coating protection of the fabricated wavelength selective reflectors.

lacquer spray, directly onto the nanostructure. These coatings help prevent physical or chemical degradation over time. While the exact thickness of the coating is not critical, it is essential to account for the change in refractive index n. Specifically, to maintain the desired optical response, the step height of the fabricated structure should be adjusted according to the relation:

$$h = \frac{\lambda_0 \cos(\alpha_0)}{2n}, \tag{S7}$$

where λ_0 is the target wavelength and α_0 is the incident angle.

References

1. Johansen, V. E., Andkjær, J. & Sigmund, O. Design of structurally colored surfaces based on scalar diffraction theory. *JOSA B* **31**, 207–217 (2014).
2. Goodman, J. W. *Introduction to Fourier optics* (Roberts and Company publishers, 2005).
3. de l'Éclairage, C. I. Commission internationale de l'eclairage proceedings (1931).
4. Smith, T. & Guild, J. The cie colorimetric standards and their use. *Transactions optical society* **33**, 73 (1931).
